# Prevalence of radiographic appendicular osteoarthritis and associated clinical signs in young dogs

**DOI:** 10.1038/s41598-024-52324-9

**Published:** 2024-02-03

**Authors:** Masataka Enomoto, Nicholas de Castro, Jonathan Hash, Andrea Thomson, Aoi Nakanishi-Hester, Erin Perry, Savannah Aker, Emily Haupt, Logan Opperman, Simon Roe, Tracey Cole, Nichola Archer Thompson, J. F. Innes, B. Duncan X. Lascelles

**Affiliations:** 1 Translational Research in Pain Program, Department of Clinical Sciences, College of Veterinary Medicine, Comparative Pain Research and Education Centre, North Carolina State University, Raleigh, NC USA; 2 Department of Statistics, North Carolina State University, Raleigh, NC USA; 3 Elanco Animal Health, Hook, London, UK; 4 Movement Independent Veterinary Referrals, Cheshire, England, UK; 5 Department of Anesthesiology, Center for Translational Pain Research, Duke University, Durham, NC USA; 6 Thurston Arthritis Center, UNC, Chapel Hill, NC USA

**Keywords:** Musculoskeletal system, Skeleton, Pain

## Abstract

This study aimed to determine the prevalence of osteoarthritis (OA) and associated clinical signs in young dogs. Owners of dogs aged 8 months–4 years from a single practice, were contacted in random order, to participate in a general health screen. Clinical and orthopedic examinations were performed. Each joint was scored for pain reactions (0–4). Orthogonal radiographs of all joints were made under sedation. Each joint was scored for radiographic OA (rOA) severity on an 11-point scale. Clinical OA (cOA) was defined as an overlap of rOA and joint pain in ≥ 1 joint. Owners completed OA questionnaires. The owners of 123 dogs agreed to participate. Overall, 39.8% (49/123) of dogs had rOA in ≥ 1 joint, and 16.3% (20/123) or 23.6% (29/123) dogs had cOA, depending on the cut-off value of joint pain; moderate (2), or mild (1), respectively. Owners of dogs with cOA observed signs of impairment in approximately 30% of cases. Only 2 dogs with cOA were receiving OA pain management. The most commonly affected joints in descending order of frequency were elbow, hip, tarsus, and stifle. Radiographically visible OA is common in young dogs, and 40–60% of dogs with rOA had cOA. However, OA-pain appears underdiagnosed and undertreated in young dogs.

No comprehensive, prospective studies of the prevalence of canine osteoarthritis (OA) throughout the skeleton have been performed and current estimates of OA prevalence pertain to older dogs. The most often quoted estimate of the number of dogs with clinical signs associated with OA is ‘20% of the population’^1^. The estimate of 20% comes from a 1997 publication that referenced Pfizer survey data pertaining to general practices^1^. A survey of admissions to US veterinary teaching hospitals over a 10-year period (1980 to 1989), using diagnoses entered into the medical records, found that 24% of all patients had been affected by a disorder of the musculoskeletal system, and 2% overall had been affected by degenerative joint disease of the appendicular skeleton OA^2^. However, data from veterinary teaching hospitals may not be reflective of general practice. Previous studies have estimated that the annual period prevalence of appendicular joint OA and associated pain was 2.5–6.6% in the UK and 6.1% in the US in dogs of any age attending primary-care practices^3–5^. In contrast, recently, employing a screening checklist in general practices, investigators found ~ 37% of dogs presenting to first opinion practices (in the US) had a diagnosis of confirmed (radiographs and clinical signs) or presumed (clinical signs; radiographs not taken) OA^6^. This is almost double the most commonly used estimate of 20% of the canine population. Although strong, comprehensive, prospective data on prevalence currently do not exist, OA in dogs is likely a very common disease.

Despite recent new information, the prevalence of OA-associated clinical signs in young dogs is completely unknown. This information is crucial to know because OA is thought to be initiated primarily by developmental disease in dogs^7^, and therefore could be argued to be a young-dog disease, and, although unexplored, early intervention at this stage may improve outcome later. However, if OA is not recognized in young dogs, the opportunity for early intervention is missed. Currently, most diagnoses of OA in dogs are made later in a dog’s life when clinical signs are more evident/overt^3^. The aim of this study was to define the prevalence of both radiographic OA, and of OA-associated pain (combination of radiographic OA and detectable joint pain) in dogs less than 4 years old.

## Results

A total of 320 owners were contacted, and owners of 123 dogs across 40 different breeds agreed to participate in the study. The final number of dogs in each age band was 25, 26, 30, and 42 respectively. Across all 123 dogs, mean (± SD) age, body weight, and body condition score were 29.8 ± 11.5 months, 24.0 ± 10.4 kg, and 4.9 ± 0.7, respectively. 16 dogs were intact male, and 53 dogs were castrated male; 6 dogs were intact female, and 48 dogs were spayed female (Supplemental file 1). The most common breeds were mixed (n = 32), Labrador Retriever (n = 12), and American Staffordshire terrier (n = 7) (for full list of breeds see Supplemental file 2).

All dogs were COAST (Canine OsteoArthritis Staging Tool) staged. Approximately 20% of the dogs were preclinical/no joint pain (COAST Stage 0 = 12/123 dogs and COAST Stage 1 = 11/123). The remainder were classed as COAST Stage 2 (70/123), 3 (27/123) or 4 (3/123) due to clinical signs that were OA or non-OA related.

**Figure 1:**
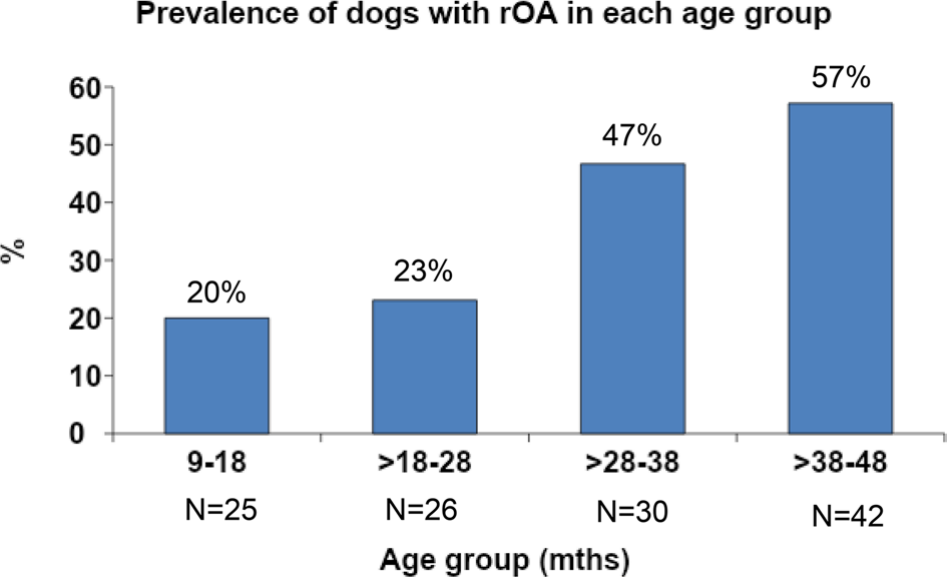
Graph depicting the prevalence of dogs with radiographic osteoarthritis (rOA) in each group. The prevalence of rOA increased with age. Overall, the prevalence of rOA was 39.8% (49/123 dogs).

Overall, 39.8% of dogs (49/123) had radiographic OA (rOA) in at least one appendicular joint. The prevalence of rOA in each age band is shown in Fig. 1 and supplemental file 3. The differences in signalment and client reported outcome measures (CROMs) scores between rOA dogs and non-rOA dogs are detailed in Table 1.

**Table 1:** Mean ± SD (median, range) values of signalment and client reported outcome measures between dogs with radiographic OA (rOA) and without rOA.

	rOA (49 dogs)	Non-rOA (74 dogs)	*p*-Value
Age	34.1 ± 10.0 (37.0, 13.0–49.0)	27.0 ± 11.7 (24.5, 9.0–49.0)	** < 0.001**
Sex	M: 4, F: 3, MC: 22, FS: 20	M: 12, F: 3, MC: 31, FS: 28	0.57
Breed	Mixed, Labrador	Mixed, Labrador	0.11
Body weight	27.4 ± 10.7 (27.4, 4.1–67.0)	21.7 ± 9.5 (23.3, 3.8–42.9)	**0.003**
BCS (1–9)	5.1 ± 0.78 (5, 4–7)	4.8 ± 0.63 (5, 4–7)	**0.017**
CBPI PSS	0.38 ± 1.0 (0, 0–4.75)	0.07 ± 0.36 (0, 0–2.75)	** < 0.001**
CBPI PIS	0.22 ± 0.68 (0, 0–4.14)	0.02 ± 0.14 (0, 0–1.14)	**0.002**
LOAD	6.1 ± 4.8 (5, 0–19)	3.9 ± 3.2 (3, 0–17)	**0.011**
SNoRE *	3.19 ± 1.5 (2.9, 1–8)	2.6 ± 0.87 (2.4, 1.4–5.4)	0.054
COAST	2.6 ± 0.6 (3, 2–4)	1.6 ± 0.9 (2, 0–4)	** < 0.001**
Total OA score	5.8 ± 5.1 (4, 1–24)	0	** < 0.001**

*M* Male, *F* Female, *MC* Male castrated, *FS* Female spayed, *rOA* radiographic osteoarthritis; *BCS* body condition score; *CBPI* Canine Brief Pain Inventory; *PSS* Pain Severity Score; *PIS* Pain Interference Score; *LOAD* Liverpool Osteoarthritis in Dogs; *SNoRE* Sleep and Nighttime Restlessness Evaluation; *COAST* Canine OsteoArthritis Staging Tool.

Significant if *p* < 0.05 (bolded).

*1 owner did not fill out SNoRE because the dog sleeps outside (OA dog).

**Figure 2:**
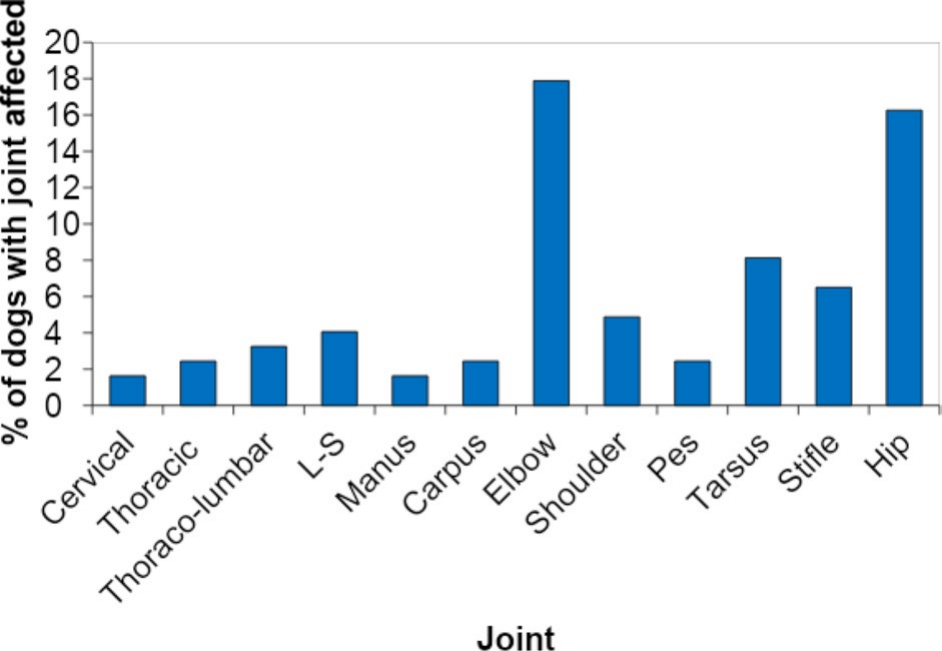
This figure shows the prevalence of radiographic osteoarthritis (rOA) across joints. The most commonly affected joints in order were elbow, hip, tarsus, and stifle.

**Figure 3:**
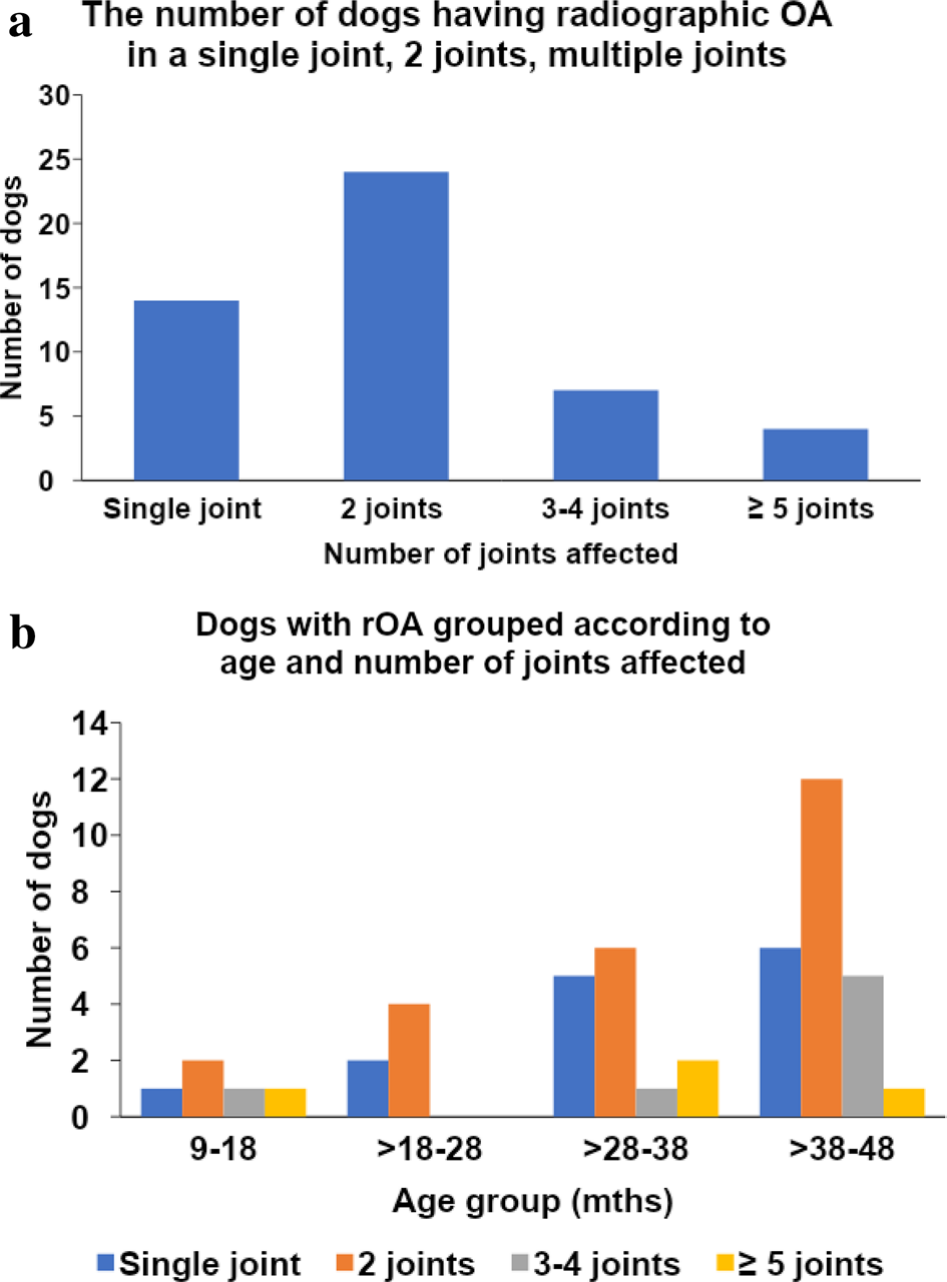
(**a**) This figure shows the number of dogs having radiographic appendicular joint osteoarthritis (rOA) in a single joint, 2 joints, and multiple joints. (**b**) This figure shows the number of dogs having rOA in a single joint, 2 joints, and multiple joints in each age group.

Radiographically affected joints in descending order of frequency were elbow, hip, tarsus, and stifle (Fig. 2). Additionally, Table 2 and Fig. 3a show how many individual appendicular joints were affected: rOA was found in 1 joint in 14 dogs; 2 joints in 24 dogs; 3–4 joints in 7 dogs; ≥ 5 joints in 4 dogs. Figure 3b shows the prevalence of dogs in each age group with varying numbers of joints affected with OA.

**Table 2:** The number of dogs having radiographic osteoarthritis (rOA) in a single joint, bilateral joints, 2 joints (different), multiple joints, and only spine.

Age category (months)	Normal (No rOA/DJD)	Single joint rOA	Bilateral joint rOA	2 joints with rOA (different joint types)	3–4 joints with rOA	≥ 5 joints with rOA	Spine DJD only
9–18	19	1	1	1	1	1	1
> 18-28	18	2	3	1	0	0	2
> 28–38	15	5	4	2	1	2	1
> 38–48	17	6	10	2	5	1	1
Total	69	14	18	6	7	4	5

Single joint rOA: rOA in one joint site and only on one side (unilateral) (e.g. left elbow OA).

Bilateral: A dog had rOA in one joint type and had rOA in both left and right joints (e.g. bilateral hips).

2 joints rOA: A dog had rOA in two different joints (no bilateral disease; e.g. right elbow and right hip).

3–4 joints rOA: A dog had rOA in 3–4 joints (e.g. elbows and hips).

≥ 5 joints rOA: A dog had rOA in 5 or more joints (e.g. elbows, hips, and stifles).

Only spine DJD: A dog had DJD in spine, but no OA in appendicular joint.

*rOA* radiographic osteoarthritis; *DJD* degenerative joint disease.

There was a significant difference in age, bodyweight, and body condition score (BCS) between non-rOA and rOA dogs (Table 1). However, prevalence of rOA was increased with age (*p* = 0.001), and bodyweight (*p* = 0.006), with these two factors being independently associated with rOA. BCS did not have significant impact on the prevalence of rOA (*p* = 0.79, 0.87, 0.34 for score of 5, 6, and 7 respectively). The number of dogs with rOA and non-rOA and that had risk factors for OA is tabulated in supplemental file 4. Three dogs had two risk factors and had OA (Breed and BCS > 7, 1 dog; breed and early neuter, 2 dogs). The relationship between the total radiographic score and age is shown in supplemental file 5.

Twenty-two toy/small breed dogs were identified in the study, and five of them had rOA (22.7%). More detailed information of toy/small breed dogs with OA is tabulated in supplemental file 6. We considered dogs to be toy/small breed if they were a breed listed on the American Kennel Club website as “toy/small breed dogs” but we also included mixed breed dogs with body weight < 30lbs (14 kg) as ‘small breeds’ (for full list of toy/small breeds see supplemental file 7).

With respect to the prevalence of OA-associated pain (‘clinical OA’, cOA), 23.6% of dogs (29/123) had cOA using a cut-off of ≥ 1 for joint pain (mild or greater pain) (designated cOA1); and 16.3% of dogs (20/123) had cOA using a cut-off of ≥ 2 for joint pain (moderate or greater pain) in at least one joint with rOA (see Tables 3 and 4) (designated cOA2).

**Table 3:** The prevalence of cOA1 and oocOA in each age group (oocOA is defined as there is overlap of radiographic OA and mild or greater joint pain (pain score ≥ 1) in the same joint, and LOAD score of ≥ 10).

Age category (months)	Total number of dogs in group	Number of dogs with OA and joint pain (cOA1)	Dogs with clinical OA and LOAD ≥ 10 (oocOA)	Dogs with clinical OA and LOAD < 10	Overall prevalence of oocOA (%) of clinical OA *and* owner detected signs of OA pain (%)
9–18	25	4	0	4	0.0
> 18–28	26	3	0	3	0.0
> 28–	30	9	3	6	10.0
> 38–48	42	13	6	7	14.3
Total	123	29	9	20	7.3

*OA* osteoarthritis, *LOAD* Liverpool OsteoArthritis in Dogs.

**Table 4:** The prevalence of cOA2 and oocOA in each age group, which is defined as there is overlap of radiographic OA and moderate or greater joint pain (pain score ≥ 2) in the same joint, and owner-assessed LOAD score of ≥ 10.

Age category (months)	Total number of dogs in group	Number of dogs with OA and joint pain (cOA2)	Dogs with clinical OA and LOAD ≥ 10 (oocOA)	Dogs with clinical OA and LOAD < 10	Overall prevalence of oocOA (%) of clinical OA *and* owner detected signs of OA pain (%)
9–18	25	4	0	4	0.0
> 18–28	26	2	0	2	0.0
> 28–38	30	6	3	3	10.0
> 38–48	42	8	3	5	7.1
Total	123	20	6	14	4.9

*OA* osteoarthritis, *LOAD* Liverpool OsteoArthritis in Dogs.

**Figure 4:**
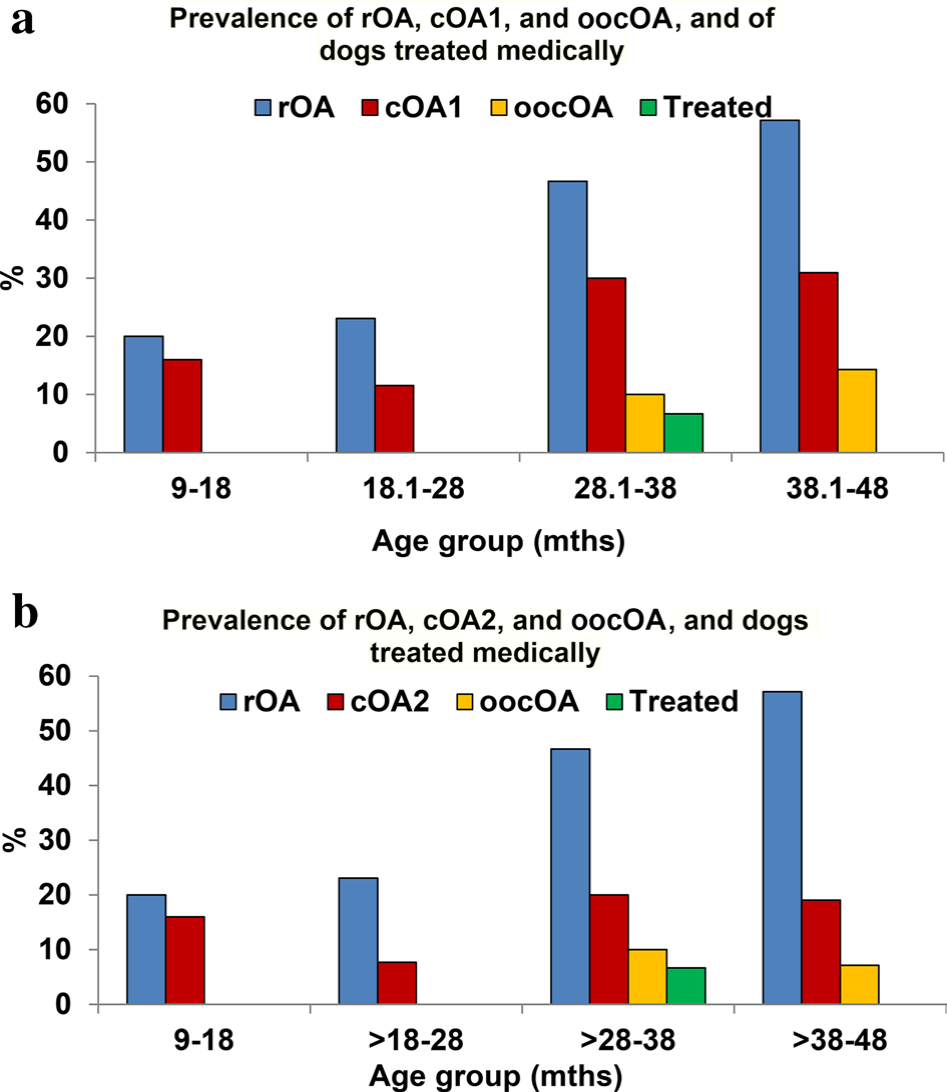
(**a**) This figure shows the prevalence of radiographic OA (rOA), clinical OA (cOA1; mild joint pain or greater), and owner-observed clinical OA (oocOA), and dogs treated medically in each age group. Blue indicates the prevalence of rOA dogs, red indicates the prevalence of cOA1, yellow indicates the prevalence of oocOA, and green indicates the prevalence of dogs being treated with pain medications at the time of screening. (**b**) This figure shows the prevalence of radiographic OA (rOA), clinical OA (cOA2; moderate joint pain or greater), and owner-observed clinical OA (oocOA), and dogs being treated medically in each age group. Blue indicates the prevalence of rOA dogs, red indicates the prevalence of cOA2, yellow indicates the prevalence of oocOA, and green indicates the prevalence of dogs treated with pain medications at the time of screening.

Based on owner completed Liverpool OsteoArthritis in dogs (LOAD) scores, owners of dogs with cOA observed signs of impairment in 9/29 (31%) for cOA1, and 6/20 (30%) for cOA2, but only 2 of them (2/9 or 2/6) were treated medically for cOA at the time of the health screen evaluation (designated ‘owner observed clinical OA, oocOA). The prevalence of rOA, cOA1, cOA2, and oocOA in each age band is shown in Tables 3 and 4, and graphically Fig. 4a,b.

Other orthopedic diseases that were detected radiographically are listed in supplemental file 8.

## Discussion

The results of this study have demonstrated that radiographically visible OA is very common in young dogs, and approximately 60% of dogs with rOA had cOA with at least mild pain (23.6% of dogs overall) in one or more joints and 40% had cOA with at least moderate pain in one or more joints (16.3% of dogs overall). In dogs with cOA, based on answers to standard OA questionnaires, owners identified the presence of clinical signs relatively infrequently (one third of dogs) and few dogs were medically treated.

No other work has explored the prevalence of radiographic OA and associated pain in young dogs. There are also relatively few studies that have evaluated the prevalence of OA in dogs of any age. Two previous studies showed that the estimated annual period prevalence of appendicular joint OA and associated clinical signs was 2.5% in the UK and 6.1% in the US in dogs of any age attending primary-care practices^3,5^. In the study performed in UK^3^, electronic patient record data from veterinary patients were reviewed to identify potential OA cases within a one-year sampling time frame from Jan 1st 2013 to Dec 31st 2013. Overall, 455,557 dogs (median age of 4.1 years) were included in the study and 16,437 candidate OA cases were identified. Of these, 6102 were reviewed in detail and 4196 dogs (2.5%) were confirmed as OA cases (newly diagnosed as OA or showed continuation of treatment for pre-existing diagnosis during the study period). Although there is no detailed description in the report from the largest general veterinary practice in the US, medical records from the more than 2.5 million dogs they cared for in 2018 were reviewed and the prevalence of clinical OA was calculated (6.1%)^5^. In another similar study,^4^ electronic patient record data were collected on 148,741 dogs attending primary attending primary care veterinary practices, and 3884 dogs (median age of 4.8 years) were randomly selected to investigate the prevalence of disorders. The study sampling frame was from Sep 1st 2009 to Mar 31st 2013. The authors reported that the prevalence of degenerative joint disease was 6.6% in dogs, however, the study did not use as tight a case definition as the aforementioned OA prevalence study in UK. The data in the literature vary greatly with respect to how many dogs are diagnosed with clinical OA but are generally surprisingly low when one considers that OA and OA-pain increase with age, and our data suggest that ~ 16% of young dogs are already displaying moderate pain or greater in at least 1 joint with rOA.

In this study, the total radiographic OA score increased with age. The slope of the trendline is not steep, but the score appears to increase mainly due to the presence of multiple joints being affected in addition to worsening of the actual OA scores (hip/elbow dysplasia). Although species are different, the trendline is not dissimilar to that seen in younger cats^8^. In the cat study, the slope of the trendline became steeper over time. We acknowledge that we do not know what the total OA scores would be in older dogs with OA, however, we anticipate a similar increase in OA burden over time (see supplemental file 9).

Our data indicate a high prevalence of rOA in young dogs, and particularly in the elbow and hip, supporting the assertion that OA is primarily driven by developmental disease in dogs. However, we did not review medical records to ascertain whether the affected joints had been diagnosed with developmental joint disease. In our study, the most commonly affected joints were elbow, hip, tarsus, and stifle. This is similar to previous findings in older dogs except for the tarsus^1,2^. It is unclear why the tarsus was not identified as a site of OA in Johnson’s study, but the population they evaluated was selected from cases sent to a teaching hospital, which might have had some selection bias.

Although the sample size of toy/small breed dogs was small, it appears that rOA is less common in toy/small dogs and also that the hip joint is the most commonly affected site in smaller breeds.

Currently, clinical OA (OA with associated joint pain) in dogs is diagnosed at a much later timepoint with more than 50% of diagnosed dogs aged from 8 to 13 years^9^. Indeed, previous work has reported that median age at first diagnosis of OA was 10.5 years in dogs attending UK primary care veterinary practices (the median age of the overall denominator population was 4.1 years)^3^. The high prevalence of disease supports the approach of actively screening younger dogs with the goal to intervene earlier and decrease the impact of OA and OA-associated pain over the dogs’ lifespan. Such a proactive approach has been suggested^10^, but has not been assessed—that is, it is not known if early intervention of some sort (e.g. strict weight management, regular low impact activity) decreases the impact of OA later in life.

In the present study, in dogs with clinical OA, owners reported (based on their answers captured via the LOAD instrument) the presence of clinical signs relatively infrequently—only one third of owners of dogs with detectable OA-pain according to the authors’ evaluations. Interestingly, regardless of cut-offs, owners did not appear to notice any signs of OA pain in dogs between 9 and 28 months (across this age range, 7 dogs had OA-pain detected on examination). There are several potential explanations for this, that are not mutually exclusive: Firstly, these younger dogs may not be displaying signs of OA-pain in the home environment (they are truly functioning well). Secondly, owners may not be recognizing the signs of OA-pain. The signs of joint pain in younger dogs do appear, clinically, to be different to that of older dogs, with an emphasis on adaptations of function rather than impaired function which is more obvious in older dogs. For example, young dogs with hip OA and pain may still be able to go on walks without tiring, still able to go up and down stairs, and still able to play, while older dogs with hip OA and pain may show obvious impairment in performing these activities. Additionally, many of the dogs were bilaterally affected, and it may be that owners are more likely to recognize clinical signs if only a single limb is affected rather than bilateral joints. Our data suggest that more joints become affected with age, and if multiple joints are affected, clinical signs may be more noticeable for owners as the impact of OA-pain becomes high. Lastly, the questionnaires used, (LOAD) and potentially other CROMs (e.g., CBPI) may not be ideal for the assessment of signs of OA-pain in younger dogs. Attempts to measure OA-pain in young dogs using CROMs may be fundamentally flawed because these instruments were developed in older dog populations. Indeed, the LOAD was developed using dogs of mean ages 7.9 years and the CBPI was developed using the dogs with > 5 years^11,12^. That said, in this study the use of LOAD identified one third of the cOA cases, suggesting that the proactive use of LOAD or other CROMs can still flag a good proportion of young dogs with cOA, and act as a starting point for conversation and facilitate the education of owners about OA. To date, there has been no comprehensive description or investigation of the behavioral signs of OA-pain in young dogs, and no attempts to develop an owner questionnaire specifically for this younger population of dogs.

Previous studies have suggested that once OA is recognized, it is perceived by both veterinarians and owners as important enough for significant clinical care, often involving long term prescription analgesics and multiple additional treatment interventions, frequent clinic visits and relatively high levels of referral^13^. Indeed, eighty-five percent of OA cases were reported to be managed with at least one clinical modality (medical or surgical treatments) following osteoarthritis diagnosis (median age of 10.5 years) and the majority of them remained on medical management^3^. Medical management is likely to be less complex earlier in the course of the disease. Earlier, effective treatment of OA-pain may better control joint pain and the longer-term negative impacts of joint pain on other dimensions such as behavioral characteristics, affective states and muscle strength^14^. However, our study showed that only a very few dogs received medical management even after clinical OA was diagnosed or in cases where owners recognized the presence of clinical signs. This likely highlights the difference in clinical signs associated with OA between younger dogs and older dogs, and possibly differences in attitudes of both veterinarians and pet owners. For example, owners may be reluctant to treat their dogs if function is not obviously impaired. However, we did not investigate the reasons for this, and this is an important area for future research.

A significant difference was observed between dogs with OA and without OA in age, body weight, and BCS in the present study. The prevalence of rOA was increased with age and bodyweight with these two factors being independently associated with rOA in this study. A recent review paper summarized the risk factors for canine appendicular OA as: genetics, breed, conformation, age, sex/neuter status, and body weight^15^. As shown in Table 1, we did not find any significant impact of sex or breed on the radiographic OA. This may be because we had a relatively small sample size and with a large wide variety of different breeds represented, the effect of breed could not be looked at in detail. The majority of dogs enrolled in the study were neutered and so the effect of sex hormones could not be evaluated. Early de-sexing has been suggested to be a risk factor for joint disease, albeit with variable influence across breeds^16^. We were not able to collect accurate data on the time of de-sexing to be able to look at that as a factor in OA status. There was a significant difference in most CROMs between dogs with OA and without OA. However, the difference was small and the score of CROMs was relatively low even in dogs with OA. This may highlight the statement above that owners may be unaware of or may have difficulty recognizing the signs of OA pain in this age group of dogs, and/or the current CROMs may not be ideal for the assessment of signs of OA-pain in younger dogs. Further, the ‘ability’ of the CROMs to detect OA-associated pain was not likely affected by treatments for OA pain because very few dogs were receiving any treatment for OA-pain.

An important limitation of our study is that clinical OA (cOA) was defined based on subjective criteria (veterinarian examination), and therefore the results may be different if the study was repeated by different investigators. There is currently no ‘gold standard’ for diagnosing or scoring severity of OA-pain in dogs, and work needs to be performed to look at the reproducibility of the sorts of subjective assessments we used. Additionally, the criteria for determining owner-observed signs of OA (LOAD) were based on criteria developed in older dogs with OA pain, thus, this may not be relevant to signs of OA in young dogs. Additional limitations of this study were that it was performed in a single practice and single geographic area, and therefore, the client and breed spectrum may not be representative of the general public/dog population in the US. For example, German shepherds make up 7% of the US dog population^17^, but only accounted for 3% of our study population. A single practice was chosen for this initial study primarily for logistical reasons around making the sample population scientifically sound. We wanted to sample in an unbiased manner—that is, have access to the full database—and using a single practice that we could work with in this manner was the best decision scientifically balanced with the cost and logistical challenges of going into multiple practices.

This study provides the foundation for increasing awareness of OA among veterinarians and dog owners with the potential to lead to earlier intervention and mitigation of the impacts of OA-pain later in life.

## Materials and methods

### Study design

The study was a cross-sectional prevalence study of radiographic and clinical OA in a stratified sample of young dogs aged 8 months to 4 years old. NC State University Institutional Animal Care and Use Committee (IACUC) reviewed and approved all methods and procedure used in the study (IACUC#19-604-O). This approval also included ethical approval. All dog owners signed a written consent form following a detailed verbal explanation of the study protocol. All methods were performed in accordance with relevant guidelines and regulations. All animal work was conducted according to the guidelines outlined in the Animal Welfare Act of 1966 and the Health Research Extension Act of 1985. This study was carried out and reported in compliance with the ARRIVE guidelines.

### Recruitment

The dogs registered in a single practice (NC State College of Veterinary Medicine Primary Care service) were grouped into 4 age bands (8–18, > 18–28, > 28–38, and > 38–48 months). Regardless of health status, the dogs in each age band were randomly ordered by using a computer software program (https://www.random.org). Then, the owners of the first 50 dogs in each group were contacted in order and asked to visit the hospital (NC State University Pain Research lab) for a ‘health screen’. The dogs in each age group whose owners were willing for them to participate in the study were included. Owners were contacted twice by email and once by phone call. If they had not responded following these attempts, the next owner/dog in sequence was contacted. Recruitment (and evaluations—see below) began mid Feb 2020 and continued through mid Aug 2021.

### Inclusion criteria

To be eligible for the study, dogs were required to be between the ages of 8 months and 4 years at the time of recruitment and ≥3.6kg body weight. Additionally, the owners were required to be willing to have their dogs examined and to have radiographs taken under sedation. There were no specific exclusion criteria.

### Examination

Physical, orthopedic, and neurologic examination, blood work, and urinalysis were performed, and data were captured. For the orthopedic examination, every joint of each limb was examined by a veterinarian experienced in evaluating canine OA (ME), and joints were graded for pain, crepitus, effusion, and thickening^18^. The manus and pes were considered as one joint region for evaluation purposes. Other appendicular joints evaluated were carpus, elbow, shoulder, tarsus, stifle, and hip. Spinal column segments were examined and graded for pain. The axial skeleton was evaluated by dividing the spine into cervical, thoracic (T1-9), thoraco-lumbar (T10-L6), and lumbosacral regions. Scores for pain ranged from 0 to 4; these scores were used to create a Total Pain Score (sum of individual pain scores for each joint) with a range of 0–64 for the appendicular skeleton. Assessments for crepitus, effusion, thickening, and range of motion were recorded, but not used in analysis. Scores were recorded on the JESSE (canine) (Supplemental file 10).

### Client reported outcome measures (CROMs)

CROMs were used as previously described^11,12,19–21^. The Liverpool Osteoarthritis in Dogs (LOAD) and Canine Brief Pain Inventory (CBPI) have been shown to be a valid measure of the impact of OA- pain in dogs^12,19,20,22^. Sleep and Nighttime Restlessness Evaluation Score Questionnaire version 2.0 (SnoRE) was used to collect the data regarding sleep quality^21^. The CROMs were completed by the dog owner. For the LOAD, the sum of each item was calculated. For CBPI (pain severity scores [PSS] and pain interference scores [PIS]) and the SNoRE, the average of each item was calculated.

### Radiography

Radiographs were taken under sedation with a mu-opiate combined with alpha-2 adrenergic agonist, for example hydromorphone 0.05–0.1 mg/kg/IV and dexmedetomidine 0.003–0.005 mg/kg/IV. However, the choice of drug and dose was adapted according to the dog’s health condition. Orthogonal views of all appendicular joints and the lateral views of the spine were taken. To minimize ionizing radiation exposure, where appropriate, radiographs were centered on the midpoint of the limb or spinal segment to reduce the number of individual exposures used. A subjective radiographic OA numerical rating scale where 0 = no radiographic abnormalities identified and 10 = most severe radiographic OA, was assigned to each joint based on presence of radiographic changes and their overall severity based on previously reported information^23^. Radiologic features considered indicative of presence of appendicular OA were osteophytes, enthesophytes, sclerosis, subchondral bone erosions and cysts. Degenerative changes on each axial segment were assessed using the same numerical scale described above (0 = no radiographic abnormalities identified, and 10 = ankylosis). Radiographic features evaluated and considered indicative of degenerative changes in the axial skeleton were osteophytes, spondylosis, disc-associated degeneration (end plate sclerosis, erosion, disc mineralization, narrowing), and subluxation. Radiographs were assessed independently using a DICOM viewer (Horos ver. 3.3.6) by two observers, a board-certified small animal veterinary surgeon (BDXL) and a veterinarian experienced in evaluating canine OA (ME)^23^. Once both individuals had scored all radiographs, scores were reviewed, and any scores that differed between reviewers were discussed a consensus score was assigned. *Note: all parts of the skeleton were assessed, but the focus of the current report is to describe appendicular OA.*

### The Canine OsteoArthritis Staging tool (COAST)

COAST was used for staging the impact of OA on patients^10^. Based on the published papers^15,24,25^, the items considered as the risk factors for OA in this study were orthopedic disease without radiographic evidence of OA (e.g. hip subluxation), traumatic joint injury/surgery, certain breed, overweight (BCS ≥ 7), and early neuter (≤ 6 months of age).

### Definitions of OA

#### Radiographic OA (rOA)

Individual dogs were defined as ‘radiographic OA (rOA)’ if the radiographic score was ≥ 1 for any appendicular joint. However, if degenerative changes were seen only in the axial skeleton (no OA in appendicular joints), the dog (as a whole) was considered as ‘non-OA’ for the purposes of this study of appendicular OA.

#### Clinical OA (cOA)

Individual dogs were defined as having ‘Clinical OA’ (cOA) using two criteria: if there was overlap of radiographic OA (score of ≥ 1) and joint pain score of ≥ 1 (mild or greater pain), they were defined at cOA1; and if there was overlap of radiographic OA (score of ≥ 1) and joint pain score of ≥ 2 (moderate or greater pain), they were defined at cOA2.

#### Owner observed clinical OA (oocOA)

Dogs were also defined as ‘Owner observed clinical OA’ (oocOA) if there was overlap of radiographic OA and joint pain in the same joint, and LOAD score was ≥ 10 (LOAD ≥ 10 is considered as ‘moderately affected’). Additionally, dogs were considered as ‘treated’ if they were receiving recognized ‘pain medication(s)’ such as non-steroid anti-inflammatories, gabapentin, amantadine, tramadol, injectable polysulfated glycosaminoglycans for the management of pain. LOAD was used to define oocOA because it has been well validated, widely used, and appears to assess the impact of joint pain multidimensionally compared to other CROMs.

#### Risk factors of rOA

Sex, age, body weight, body condition score, and breed, were compared between OA dogs and non-OA dogs to examine which factors were associated with rOA.

### Statistical analysis

Descriptive statistics were used to describe prevalence of OA and OA-associated pain. Wilcoxon rank sum tests were applied to compare the dogs with rOA and without rOA. If there was a difference, logistic regression models were built to investigate the association between the presence of OA and the difference(s). For a categorical variable with more than two levels, one variable was treated as baseline and compared with the other variables. The models considered included all combinations of the differences and their interaction terms. All statistical analyses were performed using R version 4.2.2, α = 0.05 as our cutoff for statistical significance.

### Supplementary Information

Supplementary Information 1.

Supplementary Information 2.

## Data Availability

The datasets used and/or analyzed during the current study are available from the corresponding author on reasonable request.
