# Supplementary material for: Prevalence of radiographic appendicular osteoarthritis and associated clinical signs in young dogs
Source: Sci Rep. 2024 Feb 3;14:2827. doi: 10.1038/s41598-024-52324-9 (PMC10838335; doi:10.1038/s41598-024-52324-9)
Supplement: Supplementary file 1 — Supplementary Information 1. [file 41598_2024_52324_MOESM1_ESM.docx]

**Supplemental file 1**. Mean ± SD (median, range) values of signalment and clinical metrology instruments in the dogs enrolled in the study (n=123)

|  | Mean ± SD (range) |
| --- | --- |
| Age (months) | 29.8 ± 11.5 (31.0, 9.0 - 49.0) |
| Sex | M: 16, F: 6, MC: 53, FS: 48 |
| Body weight (kg) | 24.0 ± 10.4 (23.8, 3.8 - 67.0) |
| BCS (1-9) | 4.9 ± 0.7 (5, 4 - 7) |
| COAST | 2.0 ± 0.9 (2, 0 - 4) |

BCS = body condition score; COAST = Canine Osteoarthritis Staging Tool

**Supplemental file 2**. List of breeds participated in the study (40 different breed in total across 123 dogs)

Mixed breed: 32 dogs

Labrador retriever mix: 11

Mixed: 11

American Staffordshire terrier mix: 2

Border collie mix:1

Chihuahua mix: 1

Collie mix:1

German shepherd mix: 1

Miniature pinscher mix: 1

Rottie mix: 1

Shar-pei mix: 1

Toy-poodle mix: 1

Labrador Retriever: 12 dogs

American Staffordshire terrier: 7 dogs

Golden Retriever: 6 dogs

Australian shepherd: 5 dogs

German Shepherd: 4 dogs

Hound: 4 dogs

Australian Cattle dog: 3 dogs

Boxer: 3 dogs

Collie: 3 dogs

American Bulldog: 2 dogs

Bernese Mountain dogs: 2 dogs

Catahoula Leopard dog: 2 dogs

Cavalier King Charles Spaniel: 2 dogs

Papillon: 2 dogs

Rhodesian Ridgeback: 2 dogs

Standard Poodle: 2 dogs

Airedale Terrier: 1 dog

Anatolian Shepherd: 1 dog

Beagle: 1 dog

Border Collie: 1 dog

Brittany Spaniel: 1 dog

Chihuahua: 1 dog

Doberman Pinscher: 1 dog

English Bulldog: 1 dog

English Setter: 1 dog

German Shorthaired Pointer: 1 dog

Golden Doodle: 3 dogs

Jack Russel Terrier: 1 dog

Maltese: 1 dog

Mastiff: 1 dog

Miniature Pinscher: 1 dog

Otter hound: 1 dog

Plott hound: 1 dog

Pug: 1 dog

Shar-pei: 1 dog

Shih Tzu: 1 dog

Siberian Husky: 1 dog

Toy Poodle: 1 dog

Treeing Walker Coonhound: 1 dog

**Supplemental file 3**. Prevalence of radiographic OA in each age group

| Age category | Total number of dogs in each age group | Number of dogs w/o OA | Number of dogs with OA | OA prevalence (%) |
| --- | --- | --- | --- | --- |
| 9-18months | 25 | 20 | 5 | 20.0 |
| >18-28months | 26 | 20 | 6 | 23.1 |
| >28-38months | 30 | 16 | 14 | 46.7 |
| >38-48months | 42 | 18 | 24 | 57.1 |
| Total | 123 | 74 | 49 | 39.8 |

A dog was categorized as “OA” if there was radiographic OA in at least one appendicular joint (radiographic score of ≥ 1). OA = osteoarthritis; w/o = without; % = percentage

**Supplemental file 4**. The number of dogs with OA and w/o OA under each factor

| **Risk factors** | **OA** | **Non-OA** |
| --- | --- | --- |
| BCS ≥ 7 | 4 | 1 |
| Developmental joint disease  OC/OCD  MPL  Hip subluxation w/o radiographic OA  Fragmented coronoid process  Joint instability  Cranial cruciate ligament rupture  Other appendicular joint disease  Avulsion of the long digital extensor tendon | 5  2  0  2  3  0 | 3  2  9  0  0  1 |
| Early neuter*  (≤ 6 months of age) | 9 | 13 |
| Breed  Labrador Retriever  Golden Retriever  German Shepherd  Boxer  Bernese Mountain dog  Mastiffs  Italian Corso dog | 3  3  2  1  1  1  0 | 9  3  2  2  1  0  0 |

OA: osteoarthritis, OC: osteochondrosis, OCD: osteochondritis dissecans, MPL: medial patella luxation. *No clear information was obtained in 40 dogs

**Supplemental file 5.** This figure shows the relationship between age and total radiographic score. It appears that there are two ‘humps’; the first one is 10-20 months and second one is 30-50 months. The total radiographic score increases with age (p=0.0178)

**Supplemental file 6**. Table detailing the toy/small breed dogs that had OA, the total OA score, LOAD and COAST scores, and whether the dogs were classified as only having rOA, or rOA and associated pain, or rOA, associated pain and owner-noticed signs of OA (OocOA1).

| Breed | Age (months) | BW (kg) | Affected site | OA score | LOAD | COAST | Classification |
| --- | --- | --- | --- | --- | --- | --- | --- |
| Chihuahua mix | 47 | 5.8 | Hips | 2 | 14 | 3 | OocOA |
| Papillon | 44 | 4.1 | Hip | 1 | 3 | 2 | cOA1 |
| Labrador mix | 39 | 13.2 | Hips | 8 | 0 | 3 | cOA2 |
| Labrador mix | 35 | 13.1 | Hips | 2 | 3 | 2 | rOA |
| French Bulldog | 45 | 12.1 | Manus, Pes | 3 | 2 | 2 | rOA |

OA: osteoarthritis, rOA: radiographic osteoarthritis, cOA: clinical OA, OocOA: owner observed clinical OA

**Supplemental file 7**. List of toy/small breeds participated in the study (11 different breed in total across 22 dogs)

Mixed breed small dogs: 9 dogs

Labrador mix: 3 dogs

Mixed breed: 3 dogs

Miniature Pincher mix: 1 dog

Toy poodle mix: 1 dog

Chihuahua mix: 1 dog

Cavalier king Charles spaniel: 2 dogs

French bulldog: 2 dogs

Papillon: 2 dogs

Pug: 1 dog

Beagle: 1 dog

Maltese: 1 dog

Jack Russel terrier: 1 dog

Terrier: 1 dog

Chihuahua: 1 dog

Shi Tzu: 1 dog3.

**Supplemental file 8.** The list of diseases that dogs participated in the study had.

| **Disease** | **Number of dogs** |
| --- | --- |
| Hip dysplasia w/o radiographic signs of OA | 9 (bilateral: 6, unilateral: 3) |
| OC/OCD | 8 (bilateral shoulders: 1, Unilateral shoulder: 6, unilateral tarsus: 1) |
| Medial patella luxation | 4 (Bilateral: 4) |
| Cranial cruciate ligament rupture | 3 (Bilateral: 1, unilateral: 1) |
| Fragmented coronoid process* | 2 (Bilateral: 2) |
| Avulsion of the long digital extensor tendon | 1 (Unilateral: 1) |
| Numerical anomaly of lumber vertebrae | 4 (8 lumbar: 2, 6 lumbar: 2) |
| Transitional lumbosacral junction | 1 |
| Incomplete fusion of S3-Cd1 | 1 |

OA: osteoarthritis, OC: osteochondrosis, OCD: osteochondritis dissecans. S: sacral, Cd: caudal vertebra (coccygeal) * the computed tomography or arthroscopy confirmed the disease

Supplemental file 9. The relationship between age and total radiographic score.

Ten dogs were randomly selected from the participants of another OA study (≥ 5 years old, moderate to severe OA cases defined by the client specific outcome measures ≥5) and their radiographic images were assessed using the same 11-point scale. Then, the scores were added in figure 3.
